# Retraction

**DOI:** 10.1002/ece3.7086

**Published:** 2020-12-22

Pierson, K. Building a richer understanding of diversity through causally consistent evenness measures. *Ecology and Evolution*. 2020; 10: 10965–10973. https://doi.org/10.1002/ece3.6353

The above article, published online on 26 May 2020 in Wiley Online Library (wileyonlinelibrary.com), has been retracted by agreement between the author, the journal's Editors in Chief (Allen Moore; Andrew Beckerman; Jennifer Firn; Chris Foote; Gareth Jenkins) and John Wiley & Sons Ltd. The retraction has been agreed due to the paper substantially overstating the magnitude of the problem it addresses. This has caused a change in the fundamental message of the paper.

